# Glycosite-deleted mRNA of SARS-CoV-2 spike protein as a broad-spectrum vaccine

**DOI:** 10.1073/pnas.2119995119

**Published:** 2022-02-11

**Authors:** Chung-Yi Wu, Cheng-Wei Cheng, Chih-Chuan Kung, Kuo-Shiang Liao, Jia-Tsrong Jan, Che Ma, Chi-Huey Wong

**Affiliations:** ^a^Genomics Research Center, Academia Sinica, Taipei 11529, Taiwan;; ^b^The Master Program of AI Application in Health Industry, Kaohsiung Medical University, Kaohsiung City 80708, Taiwan;; ^c^Department of Chemistry, Scripps Research, La Jolla, CA 92037

**Keywords:** COVID-19, mRNA vaccine, broad spectrum, glycosite deletion

## Abstract

Development of the messenger RNA (mRNA) vaccine has emerged as an effective and speedy strategy to control the spread of new pathogens. After vaccination, the mRNA is translated into the real protein vaccine, and there is no need to manufacture the protein in vitro. However, the fate of mRNA and its posttranslational modification inside the cell may affect immune response. Here, we showed that the mRNA vaccine of severe acute respiratory syndrome coronavirus 2 (SARS-CoV-2) spike protein with deletion of glycosites in the receptor-binding domain (RBD) or especially the subunit 2 (S2) domain to expose more conserved epitopes elicited stronger antibody and CD8^+^ T cell responses with broader protection against the alpha, beta, gamma, delta, and omicron variants, compared to the unmodified mRNA. Immunization of such mRNA resulted in accumulation of misfolded spike protein in the endoplasmic reticulum, causing the up-regulation of BiP/GRP78, XBP1, and p-eIF2α to induce cell apoptosis and strong CD8^+^ T cell response. In addition, dendritic cells (DCs) incubated with S2-glysosite deleted mRNA vaccine increased class I major histocompatibility complex (MHC I) expression. This study provides a direction for the development of broad-spectrum mRNA vaccines which may not be achieved with the use of expressed proteins as antigens.

## Significance

To contain the spread of SARS-CoV-2, the mRNA vaccine of viral spike protein has proven to be highly effective, although the efficacy against the emerging variants is decreasing. Here, we show that the mRNA of spike protein with deletion of glycosites in the RBD or especially the S2 domain to expose more conserved epitopes can be used as a broadly protective vaccine against the wild type and variants of concern. The spike protein translated from such mRNA was not properly folded, and thus induced cell apoptosis and a strong T cell response. Our findings demonstrate the importance of the glycosylation effect on the development of broadly protective mRNA vaccines.

In 1796, Edward Jenner created the first vaccine (cowpox) in the world to protect against smallpox and successfully rescued millions of people, and, since then, vaccination has been recognized as the best way to protect against pathogens (1). Since the outbreak of severe acute respiratory syndrome coronavirus 2 (SARS-CoV-2) in December 2019 that caused COVID-19, the virus has spread all over the world and caused more than 200 million infections and 4 million deaths in 20 mo (2–4). Of the various vaccines developed to control the spread of SARS-CoV-2 and variants, the messenger RNA (mRNA) vaccines developed by Moderna and BioNTech/Pfizer have proven highly effective and represent a major breakthrough in vaccine development, mainly due to the speed and convenience. These vaccines were stabilized with new mRNA technology and lipid nanoparticle (LNP) formulation for delivery and translation into the spike (S) protein in vivo to induce immune response (5–7). The nucleoside modification, sequence optimization, synthesis, and high-performance liquid chromatography purification were the major steps used for development of mRNA vaccines. However, after mRNA vaccination, the translational process and posttranslational modification of the expressed immunogen, and how they affect the outcome of immune response, are not well understood (8). Among the many posttranslational modification events, glycosylation is known to play an important role in protein folding, structure, and function (9). Our previous study showed that deletion of certain glycosites in the S protein affected the expression and folding of S protein, making it difficult to prepare pure S proteins with deletion of specific glycosylation sites for the study of their receptor binding and immune response (10). Therefore, in this study, the mRNA of SARS-CoV-2 S protein with deletion of specific glycosites is used to investigate the effect of glycosylation on protein expression and immune response in vivo.

The SARS-CoV-2 S protein has three major immunogenic domains: the N-terminal domain, the receptor-binding domain (RBD), and the subunit 2 domain (S2) (11). Previous studies have shown that the neutralizing antibodies that recognize the RBD are highly protective against SARS-CoV-2 and other coronaviruses (12–14). However, the efficacy of antibodies against the emerging variants was found to decrease as the virus continued to mutate. More than 3 million S protein sequences have been reported to the global database Global Initiative on Sharing Avian Influenza Database (GISAID), and all are highly glycosylated, with 22 *N*-glycosites and 2 O-glycosites per monomer. As part of our efforts to develop broadly protective vaccines and antibodies against SARS-CoV-2 and the emerging variants, we have shown that immunization of wild-type (WT) S protein with all 22 *N*-glycosites trimmed down to *N*-acetylglucosamine (GlcNAc) as the mono-GlcNAc decorated S protein (S_mg_) elicited broadly protective immune responses, including antibody and CD4^+^ as well as CD8^+^ T cell responses against the alpha, beta, gamma, and delta variants. Further study showed that most of the conserved epitopes on S protein are located in the RBD and the HR2 domain of the S2 subunit, but these conserved epitopes are largely shielded by glycans to escape from the immune response (10). So, removal of the shielded glycans to expose more conserved epitopes induced broader and stronger immune responses. We also used the single B cell technology to screen B cell clones from S_mg_ immunized mice, and we identified a broadly neutralizing monoclonal antibody targeting a highly conserved epitope in RBD which was not found in S_fg_ immunized mice, further demonstrating that removal of glycan shields from S protein is an effective strategy for development of a broadly protective vaccine against SARS-CoV-2 variants (10). To translate this finding into the mRNA vaccine design for development of a broad-spectrum mRNA vaccine, here we show the study of S protein mRNA vaccine with deletion of specific glycosites in RBD and S2 with N to Q and S/T to A replacement and investigation of their protein expression and immune response as well as breadth of protection.

## Result

### Glycosylation of S Protein Affected Antibody Production.

**Fig. 1.**
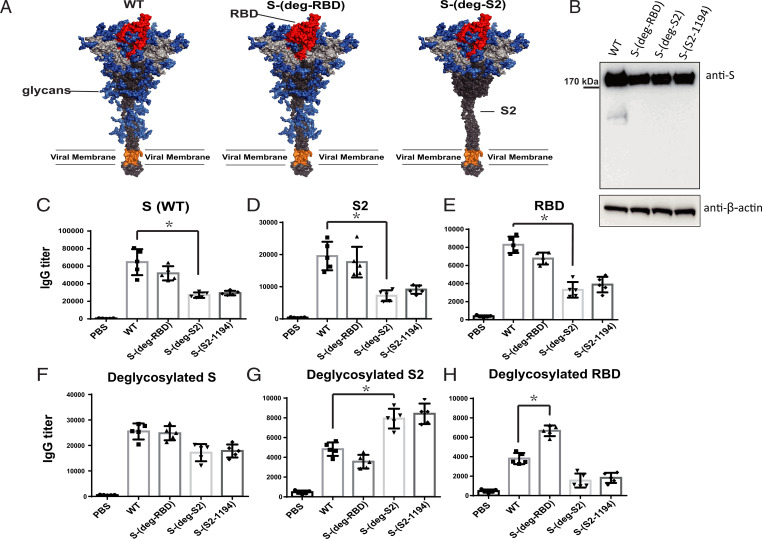
Glycosylation regulated antibody production. (*A*) A 3D image of the SARS-CoV-2 spike protein and vaccine design. Blue, glycan; red, RBD; gray, S1; dark gray, S2; orange, transmembrane domain. (*B*) Analysis of S protein expression after transfection with mRNA at 48 h by Western blot. The filter was probed with anti-S and anti–β-actin monoclonal antibodies. Humoral immune response in BALB/c mice was shown as protein-specific IgG titer from serum against (*C*) S WT, (*D*) S2, (*E*) RBD, (*F*) deglycosylated S, (*G*) deglycosylated S2, and (*H*) deglycosylated RBD, analyzed by ELISA. *C*–*H* show mean ± SD for five independent experiments. *P < 0.001.

**Fig. 2.**
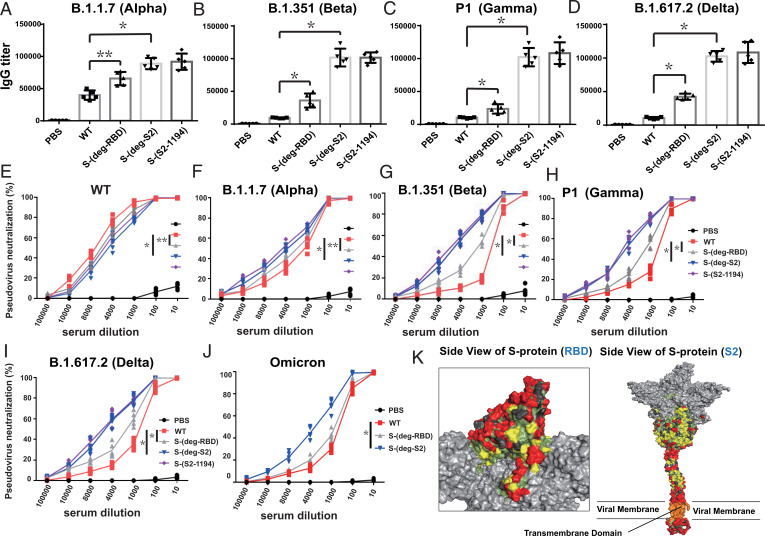
Glycosylation affected the specificity of elicited antibodies and the breadth of mRNA vaccine protection. The (*A*) alpha, (*B*) beta, (*C*) gamma, and (*D*) delta S protein–specific IgG antibody endpoint titers determined by ELISA. Neutralization curves of pseudovirus variants are shown with (*E*) WT, (*F*) alpha, (*G*) beta, (*H*) gamma, (*I*) delta, and (*J*) omicron. (*K*) The S protein 3D image of SARS-CoV-2 colored by conserved residues. Red, conserved and exposed residues (not shielded by glycans); yellow, conserved and exposed residues (shielded by glycans); green, buried residues; dark gray, mutation sites (mutation rate > 0.05%); light gray, nonemphasized regions. *A*–*J* show mean ± SD for five independent experiments. *P < 0.001, **P < 0.05.

S protein is frequently mutated and highly glycosylated (with 2 *N*- and 2 O-glycosites in RBD, 11 *N*-glycosites in S1, and 9 *N*-glycosites in S2) to evade host immune response (*SI Appendix*, Fig. S1) (15). To study how the mRNA vaccine of S protein with deleted glycosites affected S protein expression and immune response, we deleted multiple *N*-glycosites (from N to Q in the N-X-S/T sequon) and O-glycosites (from S/T to A) where the conserved epitopes in the RBD or the S2 region were shielded by the glycans (Fig. 1*A*). We previously also found that the four glycosites in RBD and the *N*-glycosite at N-801 or N-1194 in S2 were important for the integrity of S protein and its binding to the receptor. After confirming the expression of the variant prefusion S protein in the HEK293 cell line (Fig. 1*B*), the mRNA that encoded the S protein or the S protein with deletion of specific glycosites was encapsulated in LNP to form mRNA-LNP for immunization in mice. Sera from mice immunized by the mRNA with all S2 *N*-glycosites deleted (S-(deg-S2)) or with all S2 *N*-glycosites except N-1194 deleted (S-(S2-1194)), showed lower IgG titer against the fully glycosylated WT S protein (Fig. 1*C*), S2 (Fig. 1*D*), RBD (Fig. 1*E*), or deglycosylated S protein (Fig. 1*F*), but had higher IgG titer against the deglycosylated S2 antigen (Fig. 1*G*) in enzyme-linked immunosorbent assay (ELISA) as compared to the unmodified mRNA. However, the mice immunized with the mRNA with all RBD glycosites deleted (S-(deg-RBD)) elicited slightly lower IgG titer against the fully glycosylated RBD, but higher IgG titer to recognize the deglycosylated RBD antigen (Fig. 1*H*), suggesting that glycosylation on S protein affected the production of antibody and its binding specificity. Interestingly, immunization with S-(deg-RBD), S-(deg-S2) or S-(S2-1194) mRNA elicited a higher IgG titer against the alpha (Fig. 2*A*), beta (Fig. 2*B*), gamma (Fig. 2*C*), delta, and omicron variants (Fig. 2*D*), suggesting that glycosylation of S protein regulates the specificity of antibodies generated by the mRNA vaccine. To analyze the effect of glycosite deletion on the neutralization activity of antibodies generated from immunized mice, the pseudovirus neutralization assay was performed, and the result showed that the mRNA vaccine with deletion of glycosites in RBD or S2 generated antibodies with reduced neutralization activity against WT pseudovirus (Fig. 2*E*), but with better neutralization activity against the five variants of concern than WT (Fig. 2 *F*–*I* and *SI Appendix*, Table S1). This finding is consistent with our previous study of sequence alignment analysis revealing that deletion of the glycosites in RBD or S2 will expose more conserved epitopes in immune responses (Fig. 2*J*), and that the RBD and the S2 domain contained most of the highly conserved sequences in the S protein (10). Taken together, our findings suggest that deletion of specific glycosites in the mRNA vaccine will affect the specificity of antibody and T cell responses.

### Glycosylation Affected Cellular and Cytokine Response.

**Fig. 3.**
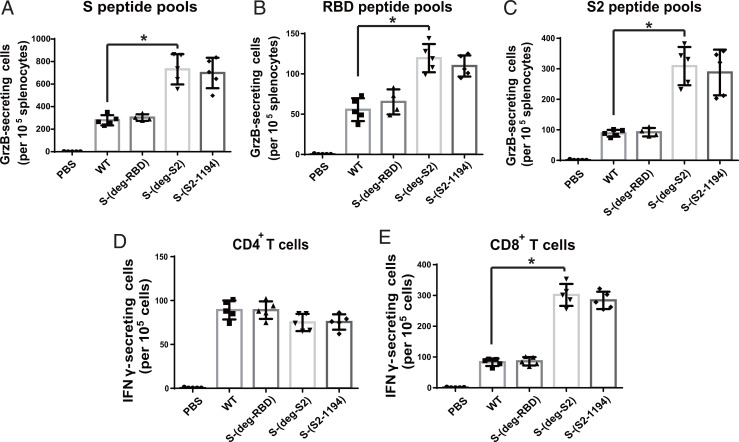
Glycosylation affected CD8^+^ T cell response. After incubation of the splenocytes isolated from immunized mice with full-length (*A*) WT S, (*B*) RBD, and (*C*) S2 peptide pools, the GrzB-secreting cells were measured by ELISpot. The (*D*) CD4^+^ and (*E*) CD8^+^ T cells were isolated and incubated with bone marrow–derived DCs and full-length WT S peptide pool to measure the IFNγ-secreting T cells by flow cytometry. *A*–*E* show mean ± SD for five independent experiments. *P < 0.001.

To characterize the T cell response, splenocytes from immunized mice were isolated and incubated with the peptide pool of S protein to measure granzyme B (GrzB)-secreting T cells by enzyme-linked immune absorbent spot (ELISpot) analysis. It was found that S-(deg-S2) and S-(S2-1194) induced more GrzB-secreting cells than WT and S-(deg-RBD) did after incubation with full-length WT S (Fig. 3*A*), RBD (Fig. 3*B*), and S2 peptides (Fig. 3*C*), suggesting that glycosylation on S2 regulated T cell response. To further study the effect on CD4^+^ and CD8^+^ T cells, the isolated T cells were incubated with bone marrow–derived dendritic cells (DCs) and WT S peptide pool to measure the IFNγ-secreting T cells by flow cytometry. The result showed that there was no significant difference in the number of IFNγ-secreting CD4^+^ T cells among all vaccinated mice (Fig. 3*D*), but the mRNA vaccine of S-(deg-S2) or S-(S2-1194) induced more IFNγ-secreting CD8^+^ T cells, suggesting that glycosylation in S2 significantly regulated CD8^+^ T cell response (Fig. 3*E*).

**Fig. 4.**
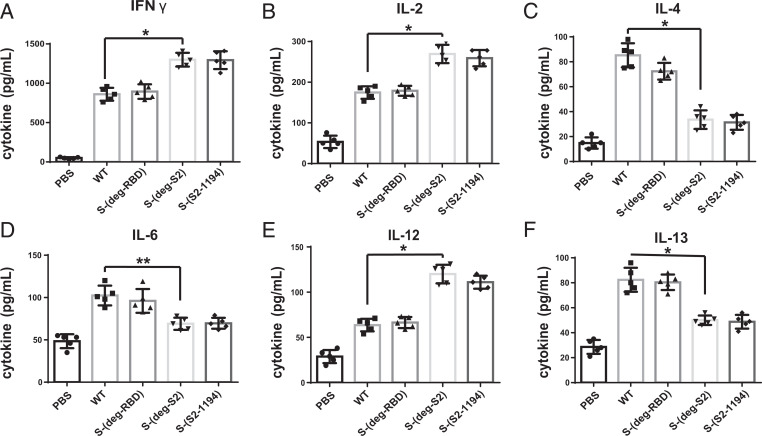
Glycosylation affects cytokines production. After incubation of the splenocytes isolated from mRNA vaccine immunized mice with full-length WT S peptide pool, (*A*) INFγ, (*B*) IL-2, (*C*) IL-4, (*D*) IL-6, (*E*) IL-12, and (*F*) IL-13 were measured. *A*–*F* show mean ± SD for five independent experiments. *P < 0.001, **P < 0.05.

To analyze cytokine expression, the medium from splenocytes incubated with full-length WT S peptide pool was measured by ELISA. It was shown that the splenocytes from S-(deg-S2) and S-(S2-1194) immunized mice secreted higher levels of T-helper-1 (TH1) cytokines (IFNγ, IL-2, and IL-12), whereas the splenocytes from WT and S-(deg-RBD) immunized mice secreted higher levels of T-helper-2 (TH2) cytokines (IL-4, IL-6, and IL-13) (Fig. 4) (16–20). Overall, all RNA vaccines with deletion of glycosites elicited antibody and CD4^+^ and CD8^+^ T cell responses and related cytokines, with a stronger IFNγ-producing CD8^+^ T cell response in mice immunized with S-(deg-S2) and S-(S2-1194). Taken together, these results suggest that glycosylation on S2 significantly regulated the T cell response and expression of cytokines.

### Deglycosylation in S2 Induced Immune Response to Unfolded Protein.

**Fig. 5.**
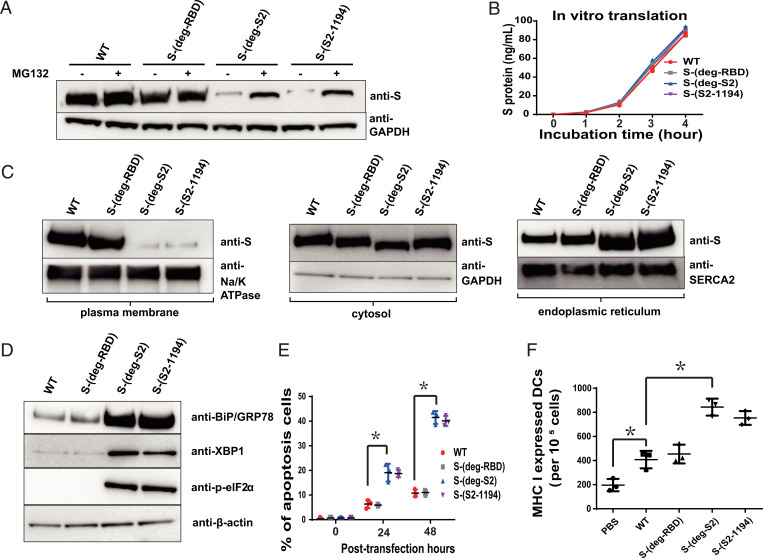
Deletion of glycosites in mRNA to produce deglycosylated S protein and the UPR. (*A*) Analysis of deglycosylated S protein expression in HEK293T cells transfected with plasmids and MG132 treatment, by Western blot. The filter was probed with anti-S and anti-GAPDH monoclonal antibodies. (*B*) In vitro translation of deglycosylated S variants at different incubation time points as shown in the figure was monitored by ELISA. (*C*) After HEK293 cells were transfected with mRNA vaccine, and after 48 h, the plasma membrane, cytosol (without ER), and ER were isolated by Western blot to analyze the amount of S protein. The filter was probed with anti-S, anti-Na/K ATPase, anti-SERCA2, and anti-GAPDH monoclonal antibodies. (*D*) Analysis of UPR marker proteins BiP/GRP78, XBP1, and p-eIF2α by Western blot 48 h after HEK293 cells were transfected with the mRNA vaccine of deglycosylated S protein variants. The filter was probed with anti-BiP, anti-XBP1, anti–p-eIF2α, and anti–β-actin monoclonal antibodies. (*E*) Analysis of cell apoptosis by APO BrdU TUNEL assay after HEK293 cells were transfected with mRNA vaccine at different time points. (*F*) Analysis of MHC I expression by flow cytometry of DCs after incubation with variants of mRNA vaccines. *B*, *E,* and *F* show mean ± SD for three independent experiments. *P < 0.001.

To investigate how glycosylation on S2 affected immune response, HEK293 cells were transfected with the prefusion stabilized S protein expression plasmid of variants. It was shown that S-(deg-S2) and S-(S2-1194) did not express well, but the expressed levels of S-(deg-S2) and S-(S2-1194) proteins were restored, to some extent, after treatment with MG132, a proteasome inhibitor (Fig. 5*A*). In addition, in vitro translation assay showed that deletion of glycosites in the mRNA sequence did not affect the efficiency of translation (Fig. 5*B*). These results suggest that removal of glycosylation in S2 affects the integrity of the translated protein in vivo. To study whether removal of glycosylation in S2 led to the expression of misfolded or unfolded protein, the plasma membrane and endoplasmic reticulum (ER) were isolated to examine the distribution of S protein, as misfolded or unfolded S proteins could be stacked in the ER for refolding or destroyed through ER-associated degradation (21). After HEK293 cells were transfected with the mRNA, and after 48 h, all variant S proteins existed in the plasma membrane, cytosol, and ER. However, more WT and S-(deg-RBD) proteins were found in the plasma membrane, whereas there was more protein of S-(deg-S2) and S-(S2-1194) in the ER (Fig. 5*C*). In addition, HEK293 cells transfected with the mRNA that encoded the soluble prefusion version (22) of S-(deg-S2) and S-(S2-1194) protein could not be secreted to the medium (*SI Appendix*, Fig. S2), suggesting that deglycosylation in S2 affected the folding of expressed S protein.

Since the increased unfolded S protein in the ER would trigger the unfolded protein response (UPR), the UPR marker proteins BiP/GRP78, XBP1, and p-eIF2α were examined in RNA transfected HEK293 cells 48 h after transfection (23–25). The results showed that BiP/GRP78 and XBP1 were up-regulated, and the level of p-eIF2α was higher in S-(deg-S2) and S-(S2-1194) transfected cells than that of the WT and S-(deg-RBD) groups (Fig. 5*D*). In addition, removal of glycosylation in S2 induced more apoptosis cells than WT and S-(deg-RBD) did (Fig. 5*E*). These results indicate that deglycosylation in the S2 domain induced more ER stress than the WT and S-(deg-RBD) protein, and affected S protein folding and expression of UPR.

### Effect of Glycosylation on MHC I and MHC II Expression on DCs.

**Fig. 6.**
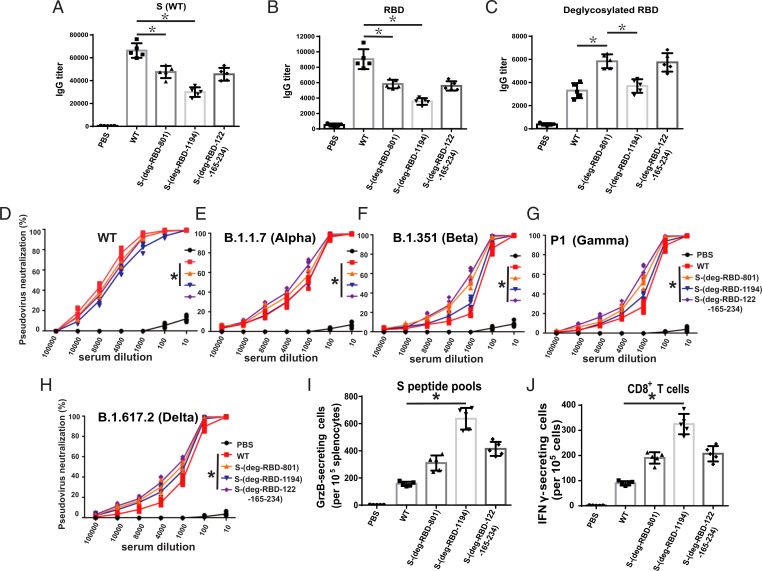
Characterization of the immune response from glycosite-deleted S mRNA vaccine. Humoral immune response in BALB/c mice was shown as protein-specific IgG titer from serum against (*A*) S WT, (*B*) RBD, and (*C*) deglycosylated RBD analyzed by ELISA. Neutralization curves of pseudovirus variants are shown with (*D*) WT, (*E*) alpha, (*F*) beta, (*G*) gamma, and (*H*) delta variants. (*I*) After incubation of the splenocytes isolated from immunized mice with full-length WT S peptide pools, the GrzB-secreting cells were measured by ELISpot. (*J*) The CD8^+^ T cells from immunized mice were isolated and incubated with bone marrow–derived DCs and full-length WT S peptide pool to measure the IFNγ-secreting T cells by flow cytometry. *A*–*J* show mean ± SD for five independent experiments. *P < 0.001.

To study whether UPR leads to the biased immune response, major histocompatibility complex class I (MHC I) and class II (MHC II) on DCs, which are essential for presentation of the internalized molecules after processing, were measured by flow cytometry (26). After DCs were incubated with variants of mRNA vaccine, MHC I/MHC II were up-regulated among all vaccines, and the mRNA vaccine of S-(deg-S2) or S-(S2-1194) induced more MHC I expression than WT and S-(deg-RBD) did (Fig. 5*F* and *SI Appendix*, Fig. S3), suggesting that UPR regulated the expression of MHC I on DCs. To further study how other glycosylation sites affected the host immune response, we used S-(deg-RBD) vaccine as a model, as it induced a better antibody neutralization activity against the four variants of concern than the WT. The glycosites in RBD and N-801 (S-(deg-RBD-801)) or N-1194 (S-(deg-RBD-1194)) were deleted, as these glycosites were important for S protein expression, especially the glycosite N-1194 which was essential for the integrity of S protein and its receptor binding (10). Since the glycosites N-122, N-165, and N-234 affected the structure of RBD and the neutralization activity of antibody, we also removed these glycosites to form the S-(deg-RBD-122-165-234) vaccine (10, 27). After HEK293 cells were transfected with the prefusion stabilized S protein expression plasmid of variants, it was shown that S-(deg-RBD-801) and S-(deg-RBD-122-165-234) did not express the S protein well, and S-(deg-RBD-1194) showed a dramatic decrease in S protein expression, but the level of protein expression was restored, to some extent, after treatment with MG132 (*SI Appendix*, Fig. S4*A*). After confirming the expression of the prefusion S protein from mRNA-LNP in HEK293 cell line (*SI Appendix*, Fig. S4*B*), mice were immunized with varied mRNA vaccines. The result showed that S-(deg-RBD-801), S-(deg-RBD-1194), and S-(deg-RBD-122-165-234) induced lower IgG titer against fully glycosylated WT S (Fig. 6*A*) and RBD protein (Fig. 6*B*), but S-(deg-RBD-801) and S-(deg-RBD-122-165-234) induced a higher IgG titer against the deglycosylated RBD antigen (Fig. 6*C*) in ELISA as compared to the unmodified mRNA, suggesting that glycosylation on these glycosites regulated antibody production. To assess the neutralization activity of antibodies generated from immunized mice using pseudovirus neutralization assay, it was shown that the S-(deg-RBD-801), S-(deg-RBD-1194), and S-(deg-RBD-122-165-234) mRNA vaccines generated antibodies with reduced neutralization activity against WT (Fig. 6*D*), but with better neutralization activity against the four variants of concern (Fig. 6 *E*–*H* and *SI Appendix*, Table S2).

To characterize the T cell response, the splenocytes of immunized mice were incubated with the peptide pool of S, RBD, and S2 protein, and then the granzyme B (GrzB)-secreting T cells were measured by ELISpot analysis. It was shown that S-(deg-RBD-801), S-(deg-RBD-122-165-234), and especially S-(deg-RBD-1194) induced more GrzB-secreting T cells than WT did in all peptide pools (Fig. 6*I* and *SI Appendix*, Fig. S5 *A* and *B*). After the isolated CD4^+^ and CD8^+^ T cells were incubated with bone marrow–derived DCs and WT S peptide pool to measure the IFNγ-secreting T cells by flow cytometry, it was found that there was no significant difference in the number of IFNγ-secreting CD4^+^ T cells (*SI Appendix*, Fig. S5*C*), but the S-(deg-RBD-801), S-(deg-RBD-1194), and S-(deg-RBD-122-165-234) mRNA vaccines induced more IFNγ-secreting CD8^+^ T cells (Fig. 6*J*). Overall, the S-(deg-RBD-801), S-(deg-RBD-1194), and S-(deg-RBD-122-165-234) variants had a decreased level of protein expression in plasmid and induced a less antibody response, but these variants induced an increased level of CD8^+^ T cell response. Taken together, we have shown that the glycosylation at the glycosites associated with the folding or stability of S protein has significant impact on CD8^+^ T cell immune response.

## Discussion

The most conserved regions of SARS-CoV-2 S protein are located in the RBD and S2 domains, which were largely shielded by glycans (10), and the antibodies that recognized these conserved regions could provide a broad protection against variants of SARS-CoV-2 (13, 14). Using the mRNA technology to remove the glycan shields to better expose the conserved regions is an effective strategy of broad-spectrum vaccine design.

Interestingly, removing the glycosylation sites in the mRNA sequence to produce the target protein immunogen also changed the translational process and immune response. Among the many mRNA vaccines with deletion of specific glycosites prepared for the study, the one with deletion of the glycosites in S2 (S-(deg-S2)) is the most protective against the five variants of concern with regard to antibody neutralization activity and CD8^+^ T cell response as compared to the WT mRNA vaccine. The mRNA vaccine with deletion of glycosites in RBD (S-(deg-RBD)) also showed a stronger CD8^+^ T cell response and a higher anti-S IgG titer against the five variants of concern than the WT mRNA vaccine, although the anti-S IgG titer against the WT strain is lower. The expression of S protein with deglycosylated S2 was shown to have a decreased level in the plasma membrane and an increased level of S protein expression in the ER, but the antibodies generated are more effective against the five variants of concern, although the level of antibody production was lower than that of WT (28–30). In addition, deletion of glycans on S2 led to the accumulation of misfolded S protein in the ER and induction of UPR to trigger cell apoptosis and up-regulation of CD8^+^ T cell response. UPR is known to play an important role in virus infection and host immune response (31). The UPR marker XBP1 is essential for surface presentation of MHC I in DCs and activation of CD8^+^ T cell response to virus infection (32–34). It was shown that, during the infection of influenza A virus, HIV, or coronavirus, the other UPR markers BiP/GRP78 and p-eIF2α also affected host immune response and virus life cycle (35–38). In addition, induced cell death was found to be the key regulator for DCs to express MHC I and prime CD8^+^ T cell response (26). Therefore, we believe that removing the glycosylation sites that are important for S protein folding at the mRNA level will stimulate more CD8^+^ T cell response, and this approach provides an alternative direction toward the development of broad-spectrum vaccines.

Glycosylation on the target antigens or pathogens affects antibody response (9), but how glycosylation affects T cell response is still unknown. It is difficult or impossible to express and purify the S protein with deletion of certain glycosylation sites that affect protein folding for immunization study (10). In this study, we found that, using mRNA as a vaccine, we are able to address this problem and study the impact of protein glycosylation on immune response through expression of deglycosylated protein immunogen inside the cell.

In summary, we have developed a method, as demonstrated in this study, to address the impact of glycosylation on S protein folding and immune response in vitro and in vivo using the mRNA of S protein as an immunogen with deletion of specific glycosites. This strategy is especially useful for the study of the glycosylation sites which are essential for protein folding, because the target protein cannot be easily obtained and purified in vitro for such study. We have also demonstrated the development of broad-spectrum vaccines using the mRNA of S protein with deletion of specific glycosites to expose the conserved epitopes.

## Materials and Methods

### Cell Lines.

Human embryonic kidney cells (HEK293) were maintained in Dulbecco's modified Eagle's medium (DMEM) (lnvitrogen) with 10% heat-inactivated fetal bovine serum (FBS) (Thermo Scientific) and antibiotics (100 U/mL penicillin G and 100 gm/mL streptomycin).

### Antibodies and Proteins.

The rabbit anti–SARS-CoV-2 S polyclonal antibody, and SARS-CoV-2 full-length S, S2, RBD, and variant proteins (293T cell expressed), were purchased from Sino Biologicals. Mouse monoclonal anti–β-actin, glyceraldehyde-3-phosphate dehydrogenase (GAPDH), and rabbit monoclonal anti-MHC II antibodies were purchased from Millipore. The rabbit monoclonal anti-Na/K ATPase was obtained from ABcam. The mouse monoclonal anti-SERCA2 and rabbit monoclonal anti-MHC I antibodies were obtained from Invitrogen. The rabbit monoclonal anti-BiP/GRP78, anti-XBP1, and anti–p-eIF2α antibodies were purchased from ABclonal. All commercial antibodies were validated for specificity by companies and us via Western blot. To obtain the deglycosylated protein, S, RBD, or S2 protein was deglycosylated in a buffer solution with PNGase F (Sigma) at 37 °C for 24 h in the dark. After deglycosylation, samples were purified and checked by Western blot (9).

### mRNA Vaccine of Deglycosylated S Protein and Formulation.

The prefusion state of the S, the codon-optimized S gene of SARS-CoV-2, was synthesized by GenScript and cloned into pcDNA3.1 or pVax, and was stabilized by proline substitutions at positions K968 and V969 (S-2P) (39). The soluble version of S ended with glutamine Q1208 of S-2P followed by a T4 fibritin (foldon) trimerization motif, thrombin cleavage site, and 6xHis tag at the C terminus was constructed (22). To delete the *N*-glycosites, the putative sequon N-X-S/T was changed to Q-X-S/T and the O-glycosite was changed from S/T to A by using site-directed mutagenesis on the S-2P expression plasmid. To obtain the mRNA vaccine, the linear DNA that contained the T7 promoter, 5′ untranslated region, 3′ untranslated region, S-2P, and poly(A) tail signal sequence was amplified by using TOOLS Ultra High Fidelity DNA Polymerase (BIOTOOLS Co., Ltd.) with 1 μL of the DNA template in an mMESSAGE mMACHINE Kit (Thermo Scientific) at 37 °C for 1 h according to the manufacturer’s protocol. The mRNA was purified by RNA cleanup kit (BioLabs) according to the manufacturer’s protocol and stored at −80 °C until further use. For the formulation of mRNA-LNP, the mRNA was encapsulated in LNP using a self-assembly process in which an aqueous solution of mRNA at pH 4.0 was rapidly mixed with an ethanolic lipid mixture containing ionizable cationic lipid, phosphatidylcholine, cholesterol, and polyethylene glycol lipid (40). The compositions of LNP were 1,2-distearoyl-*sn*-glycero-3-phosphocholine (DSPC) (Sigma), cholesterol (Sigma), 1,2-dioleoyl-3-trimethylammonium-propane (DOTAP) (Sigma), and 1,2-Dimyristoyl-rac-glycero-3-methoxypolyethylene glycol-2000 (DMG-PEG 2000) (Sigma). The mRNA-LNP was characterized and subsequently stored at −80 °C at a concentration of 1 mg/mL. After HEK293 cells were transfected with 10 μg of mRNA-LNP in six wells of a plate for 48 h, the total cell lysate was collected to monitor the expression of S by Western blot.

### Animals and Immunizations.

BALB/c mice aged 6 to 8 wk old (*n* = 5) were immunized intramuscularly with 50 μg of mRNA-LNP in phosphate-buffered saline (PBS) with 300 mM sucrose. Animals were immunized at week 0 and boosted with a second vaccination at week 2, and serum samples and spleens were collected from each mouse 1 wk after the booster immunization. The animal experiments were evaluated and approved by the Institutional Animal Care and Use Committee of Academia Sinica.

### Serum IgG Titer Measure.

Anti-S protein ELISA was used to determine IgG titer. Plates were coated with 50 ng per well of variant S protein as shown in Figs. 1 and 2, and then blocked with 5% skim milk. The serum from immunized mice and horseradish peroxidase–conjugated secondary antibody were sequentially added. Peroxidase substrate solution (TMB) and 1M H_2_SO_4_ stop solution were used, and absorbance (optical density 450 nm) was read by a microplate reader.

### Pseudovirus Neutralization Assay.

Pseudovirus was constructed by the RNAi Core Facility at Academia Sinica using a procedure similar to that described previously (10). Briefly, the pseudotyped lentivirus carrying SARS-CoV-2 S protein or variant was generated by transiently transfecting HEK-293T cells with pCMV-ΔR8.91, pLAS2w.Fluc, Ppuro, and pcDNA3.1-nCoV-SΔ18. HEK-293T cells were seeded 1 d before transfection followed by delivery of plasmids into cells by TransITR-LT1 transfection reagent (Mirus). The culture medium was refreshed at 16 h and harvested at 48 and 72 h posttransfection. Cell debris was removed by centrifugation, and the supernatant was passed through a 0.45-μm syringe filter (Pall Corporation). The pseudotyped lentivirus was then stored at −80 °C. To estimate the lentiviral titer by AlarmaBlue assay (Thermo Scientific), the transduction unit (TU) of pseudotyped lentivirus was estimated by using cell viability assay. HEK-293T cells expressing human ACE2 gene were plated on a 96-well plate 1 d before lentivirus transduction. To determine the titer of pseudotyped lentivirus, different amounts of lentivirus were added into the cell culture containing polybrene (final concentration 8 μg/mL) (Sigma), and spin infection was carried out at 1,100 × *g* in a 96-well plate for 30 min at 37 °C. After incubation for 16 h, the culture medium was removed and replaced with fresh complete DMEM containing 2.5 μg/mL puromycin (Sigma). After treating the mixture with puromycin for 48 h, the culture medium was removed, and the cell viability was detected by using AlarmaBlue reagents according to the manufacturer’s instructions. The survival rate of uninfected cells was set as 100%, and the virus titer was determined by plotting the survival cells versus diluted viral dose.

For neutralization assay, heat-inactivated sera or antibodies were serially diluted and incubated with 1,000 TU of SARS-CoV-2 pseudotyped lentivirus in DMEM for 1 h at 37 °C. The mixture was then inoculated with 10,000 HEK-293T cells stably expressing human ACE2 gene in a 96-well plate. The culture medium was replaced with fresh complete DMEM (supplemented with 10% FBS and 100 U/mL penicillin/streptomycin) at 16 h postinfection and continuously cultured for another 48 h. The expression level of luciferase gene was determined by using Bright-Glo Luciferase Assay System (Promega). The relative light unit (RLU) was detected by Tecan i-control (Infinite 500). The percentage of inhibition was calculated as the ratio of RLU reduction in the presence of diluted serum to the RLU value of no serum control using the formula (RLU ^control^ − RLU ^Serum^)/RLU ^control^ (10).

### Informatic Analysis of SARS-CoV-2 S Protein.

The 1,117,474 S protein sequences of SARS-CoV-2 and their variants were extracted from the GISAID (version: April 18, 2021). The S-protein three-dimensional (3D) structure model with representative glycan profile was constructed by CHARMM-GUI and OpenMM programs. The transmembrane region of the spike protein defined by UniProt was used in this study. The input of CHARMM-GUI includes the Protein Data Bank file 6VSB_1_1_1, the representative glycan profile, and parameter settings. Relative solvent accessibility (RSA) of the spike protein with and without representative glycans is calculated by the FreeSASA program. The probe radius 7.2Å was used in the FreeSASA program to mimic the average size of the hypervariable loops in the complementarity determining region of the antibody. The RSA value of each residue used in this study was the average RSA value from three protein chains. The definition of exposed/buried residues was the same as in the study by Kajander et al. (41).

### Measurement of GrzB- and IFNγ-Secreting Cells.

A total of 5 ×10^5^ splenocytes from immunized mice were ex vivo restimulated with full-length S, RBD, and S2 peptide mix (0.1 μg/mL final concentration per peptide) (Sino Biologicals) in the GrzB ELISpot assays (R&D Systems) according to the manufacturer’s instructions, and spots were counted. For T cell subtyping, CD8^+^ T cells and CD4^+^ T cells were isolated from splenocyte suspensions using Dynabeads Untouched Mouse CD4 and CD8 Cells kit (Invitrogen) according to the manufacturer’s instructions. CD4^+^ or CD8^+^T cells (1 × 10^5^) were subsequently restimulated with 5 × 10^4^ syngeneic bone marrow–derived DCs loaded with full-length WT S peptide mix (0.1 μg/mL final concentration) (Sino Biologicals). The purity of isolated T cell subsets was determined by flow cytometry to calculate the spot counts per 1 × 10^5^ CD4^+^ or CD8^+^ T cells. For flow cytometry, cells were suspended in FACS buffer (2% (vol/vol) FBS in PBS) at a density of 10^6^ cells/mL, and the antibody used in this study was anti-IFNγ (Abcam). Cellular fluorescence intensity was analyzed by FACS Canto (BD Biosciences) and FCS Express 3.0 software.

### Measurement of IFNγ and Other Cytokines.

IFNγ, IL-2, IL-4, IL-6, IL-12, and IL-13 were measured by using the ELISA kit according to the manufacturer’s protocol (IFN-γ: Boster Biological Technology Co., Ltd; IL-2, IL-4, IL-6, IL-12, and IL-13: R&D Systems).

### DNA Plasmid Transfection and MG132 Treatment.

After HEK293 cells were seeded in a six-well plate, cells were transfected with 3 μg of plasmid by TransIT-LT1 Transfection Reagent (Mirus) and then incubated with 1 μM MG-132 (MedChemExpress) or DMSO at 37 °C for 24 h. The total lysate was collected, and the variant S expression was analyzed by Western blot.

### In Vitro Translation.

The in vitro translation was performed with the plasmid that encoded S-2P using Glycoprotein Expression in a Human IVT System (Thermo) according to the manufacturer’s instructions. The expression of S protein in different incubation time periods was monitored by SARS-COV-2 spike protein ELISA kit (ABclonal) according to the manufacturer’s protocol.

### UPR Detection.

After HEK293 cells were transfected with 10 μg of S mRNA with TransIT-mRNA Transfection Kit (Mirus) for 48 h, the plasma membrane and ER were isolated by Minute ER Enrichment Kit (Invent Biotech) according to the manufacturer’s protocol. The S protein in the plasma membrane, cytosol, and ER was analyzed by Western blot. Total lysate was collected, and the UPR markers XBP1, BiP/GRP78, and p-eIF2α were monitored by Western blot. The apoptosis cells were measured by APO-BrdU TUNEL Assay Kit (Thermo) according to the manufacturer’s instructions.

### The Soluble Version of S-2P Expression.

HEK293 cells were transfected with 10 μg of mRNA that encoded the soluble version of variant S-2P with TransIT-mRNA Transfection Kit (Mirus) for 72 h; the S protein was purified from the supernatant using Ni-nickel-nitrilotriacetic acid (NTA) affinity column (GE Healthcare). The purified protein and total lysate were analyzed by Western blot to determine the protein level of S.

### mRNA Vaccine Induced MHC I/MHC II Expression on DCs.

DCs were isolated from mice by using M-pluriBead Cell Separation kit (PluriSelect) following the procedure from the company and incubated with 10 μg of mRNA-LNP in DC culture medium (Roswell Park Memorial Institute 1640 supplemented with 20 ng/mL murine granulocyte-macrophage colony-stimulating factor [R&D Systems], 10% FBS, 50 μM 2-ME, 100 units/mL penicillin, and 100 μg/mL streptomycin) at 37 °C for 48 h, then analyzed for MHC I and MHC II expression by flow cytometry.

### Statistics and Reproducibility.

All data were presented as means ± SEM. The numbers of sample and replicates of experiments were shown as mentioned in the figure legends. Comparisons between groups were determined using Student’s *t* test. Differences were considered significant at *P < 0.001, **P < 0.05. All data were analyzed using GraphPad Prism 6 software.

## Supplementary Material

Supplementary File

## Data Availability

All study data are included in the article and *SI Appendix*.
