# Supplementary material for: Glycosite-deleted mRNA of SARS-CoV-2 spike protein as a broad-spectrum vaccine
Source: Proc Natl Acad Sci U S A. 2022 Feb 11;119(9):e2119995119. doi: 10.1073/pnas.2119995119 (PMC8892489; doi:10.1073/pnas.2119995119)
Supplement: Supplementary File [file pnas.2119995119.sapp.pdf]

Supporting Information

Glycosite-deleted mRNA of SARS-CoV-2 Spike Protein as Broad-Spectrum Vaccine

Chung-Yi Wu[a], Cheng-Wei Cheng[a,b], Chih-Chuan Kung[a], Kuo-Shiang Liao[a], Jia-Tsrong Jan[a], Che Ma[a], Chi-Huey Wong[a,c,1]

[a]Genomics Research Center, Academia Sinica, Taipei 115, Taiwan;

[b]The Master Program of AI Application in Health Industry, Kaohsiung Medical University, Kaohsiung City 80708, Taiwan; and

[c]Department of Chemistry, Scripps Research, La Jolla, CA 92037

[1]To whom correspondence should be addressed, Email: wong@scripps.edu.

Figures

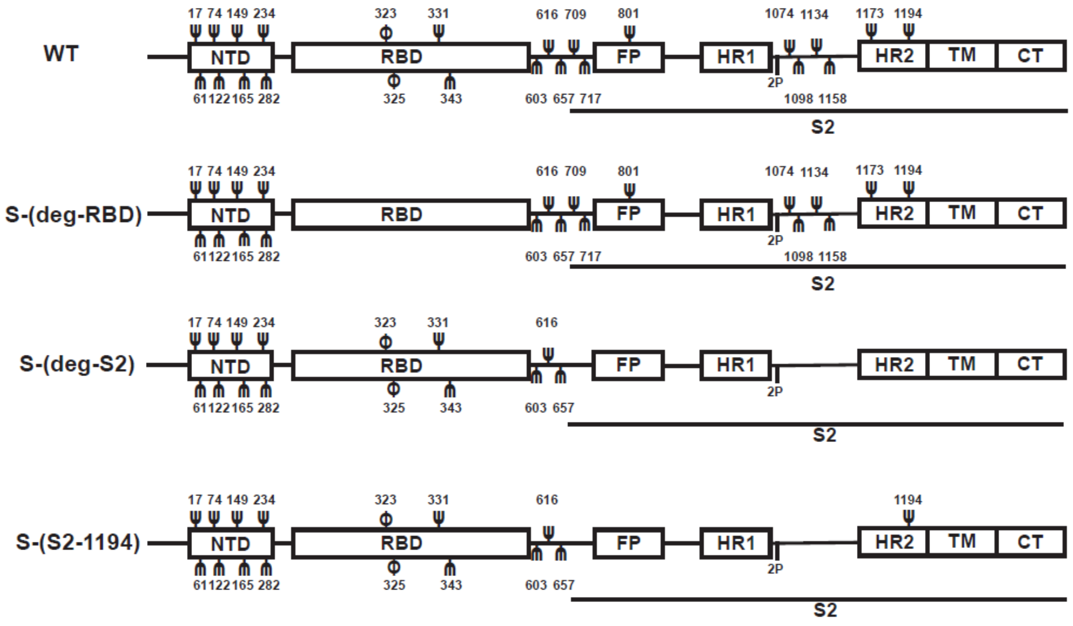

Fig S1. Schematic representation of the SARS-CoV-2 spike and vaccine design. NTD, N-terminal domain (14–305 residues). RBD, a receptor-binding domain (319–541 residues). FP, the fusion peptide (788–806 residues). HR1, heptapeptide repeat sequence 1 (912–984 residues). HR2, heptapeptide repeat sequence 2 (1163–1213 residues). TM, transmembrane domain (1213–1237 residues). CT, cytoplasm domain (1237–1273 residues). S2 subunit (686–1273 residues). 2P, (K986P, and V987P). Ψ, the N-glycosylation site; φ, O-glycosylation site.

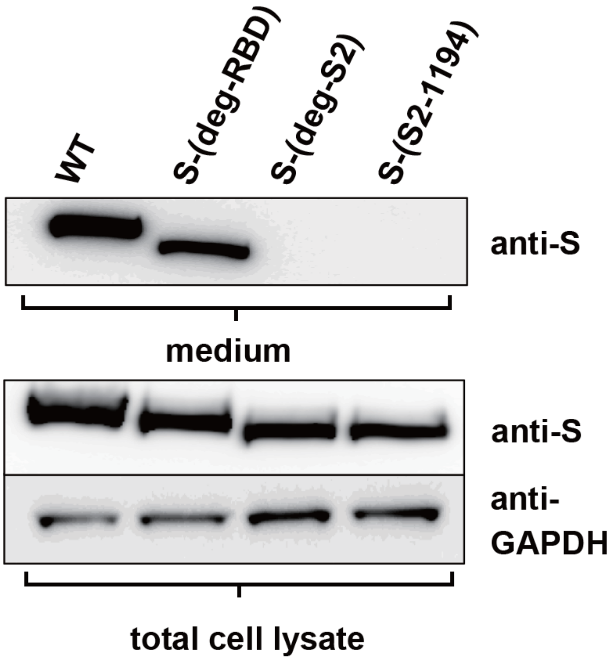

Fig S2. Glycosylation on S2 regulated the secretion of soluble pre-fusion SARS-CoV-2 spike protein. After HEK293 cells transfected with the mRNA vaccine that encoded the soluble pre-fusion version of variant S, the location of S was determined by western blot. The filter was probed with anti-S and anti-GAPDH monoclonal antibodies.

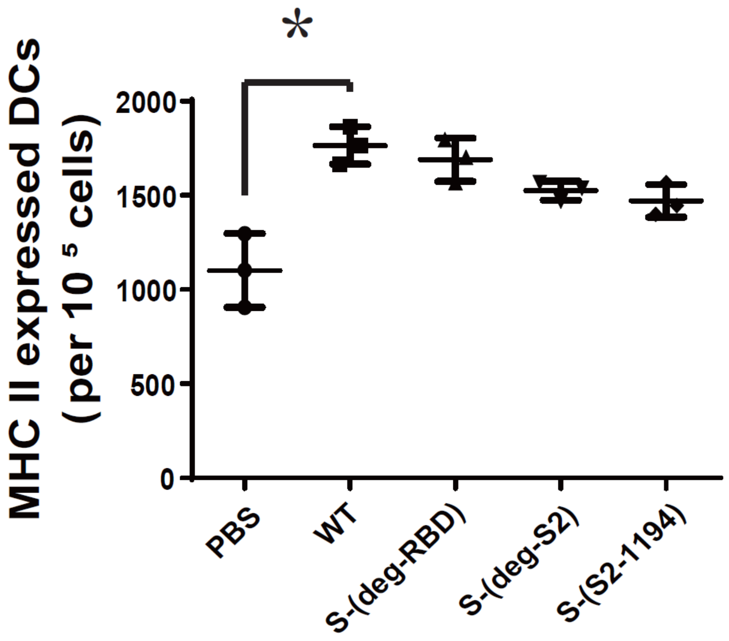

Fig S3. mRNA vaccine affected MHC II expression on DCs. Analysis of MHC II expression by flow cytometry of DCs after incubation with variants of mRNA vaccines. Mean ± SD for three independent experiments. *P < 0.001.

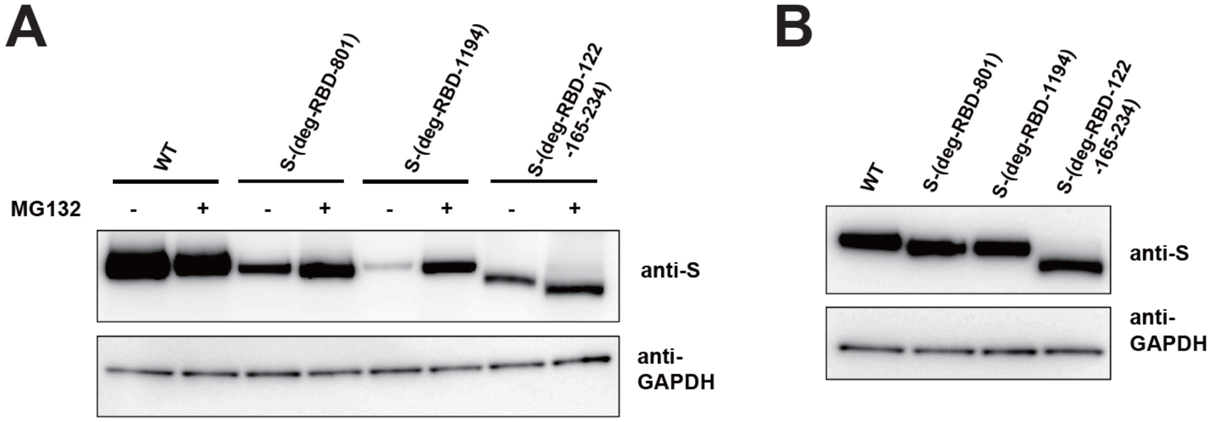

Fig. S4. The protein expression level of specific glycosites-defected S. (*A*) Analysis of various S protein expression via HEK293T cells transfected with plasmids and MG132 treatment by western blot. (*B*) Analysis of S protein expression in HEK293T cells after transfection with mRNA-LNP at 48 hr by western blot. The filter was probed with anti-S and anti-GAPDH monoclonal antibodies.

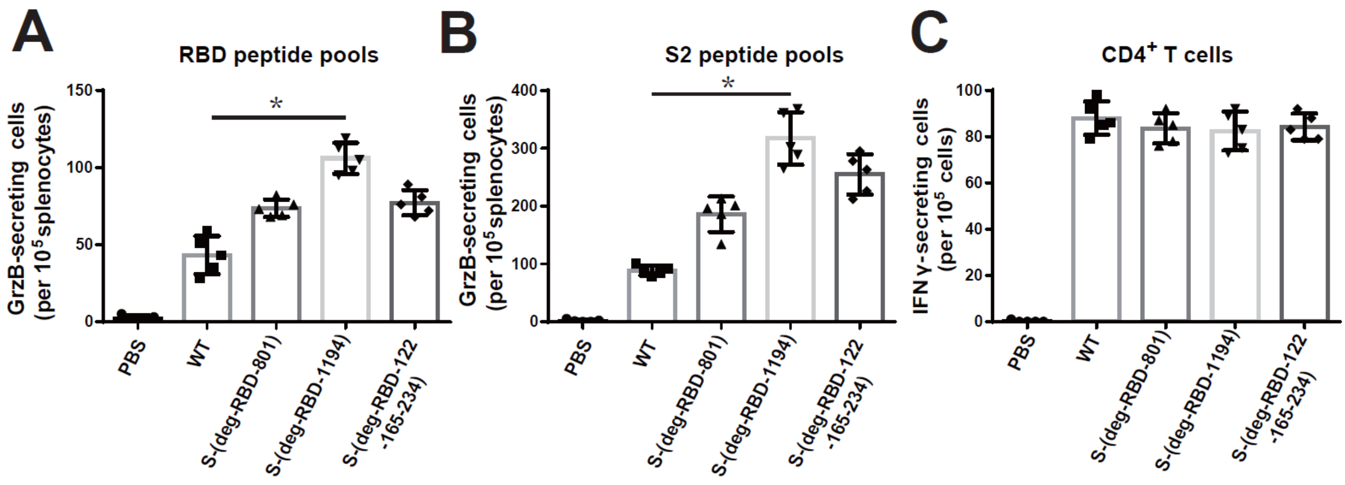

Fig. S5. Characterization of the immune response from specific glycosites-defected S mRNA vaccine. After incubation of the splenocytes isolated from immunized mice with RBD (*A*) and S2 (*B*) peptide pools, the GrzB-secreting cells were measured by Elispot. (*C*) The $CD4^{+}$ T cells were isolated from immunized mice and incubated with bone-marrow-derived DCs and full-length WT S peptide pool to measure the IFNγ-secreting T cells by flow cytometrty. (*A*-*C*) Mean ± SD for five independent experiments. *P < 0.001.

Table S1.The IG_{50} for variant pseudovirus neutralization assay.

**IC_{50}** serum dilution	WT	B.1.1.7	B.1.351	P1	B.1.617.2
WT	7658.4	1250.1	710.6	776.5	835.7
S-(deg-RBD)	6496.2	1926.5	1108.3	1523.6	1868.3
S-(deg-S2)	4825.6	4035.9	4568.3	4235.3	4786.3
S-(S2-1194)	5767.8	4628.6	5287.5	4989.6	5103.4

Table S2.The IG_{50} for variant pseudovirus neutralization assay.

**IC_{50}** serum dilution	WT	B.1.1.7	B.1.351	P1	B.1.617.2
WT	7430.8	1024.5	680.4	790.4	880.4
S-(deg-RBD-801)	5020.6	2680.4	1098.5	1640.5	1850.5
S-(deg-RBD-1194)	3480.5	1120.5	780.5	910.6	1040.3
S-(deg-RBD-122-165-234)	5240.8	3168.8	1820.8	2140.5	2040.5
